# Dynamic Arterial Elastance During Experimental Endotoxic Septic Shock: A Potential Marker of Cardiovascular Efficiency

**DOI:** 10.3389/fphys.2020.562824

**Published:** 2020-10-02

**Authors:** Manuel Ignacio Monge Garcia, Pedro Guijo González, Paula Saludes Orduña, Manuel Gracia Romero, Anselmo Gil Cano, Antonio Messina, Andrew Rhodes, Maurizio Cecconi

**Affiliations:** ^1^Unidad de Gestión Clínica de Cuidados Intensivos, Hospital Universitario SAS de Jerez, Jerez de la Frontera, Spain; ^2^Department of Intensive Care Medicine, St. George’s Healthcare NHS Trust and St George’s University of London, London, United Kingdom; ^3^Department of Anesthesia and Intensive Care Medicine, Humanitas Clinical and Research Center – Istituto di Ricovero e Cura a Carattere Scientifico (IRCCS), Rozzano, Italy

**Keywords:** cardiac output, arterial pressure, septic shock, dynamic arterial elastance, stroke volume variation, pulse pressure variation

## Abstract

Dynamic arterial elastance (Ea_dyn_), the ratio between pulse pressure variation (PPV) and stroke volume variation (SVV), has been suggested as a dynamic parameter relating pressure and flow. We aimed to determine the effects of endotoxic septic shock and hemodynamic resuscitation on Ea_dyn_ in an experimental study in 18 New Zealand rabbits. Animals received placebo (SHAM, *n* = 6) or intravenous lipopolysaccharide (E. Coli 055:B5, 1 mg⋅kg**^–^**^1^) with or without (EDX-R, *n* = 6; EDX, *n* = 6) hemodynamic resuscitation (fluid bolus of 20 ml⋅kg**^–^**^1^ and norepinephrine for restoring mean arterial pressure). Continuous arterial pressure and aortic blood flow measurements were obtained simultaneously. Cardiovascular efficiency was evaluated by the oscillatory power fraction [%Osc: oscillatory work/left ventricular (LV) total work] and the energy efficiency ratio (EER = LV total work/cardiac output). Ea_dyn_ increased in septic animals (from 0.73 to 1.70; *p* = 0.012) and dropped after hemodynamic resuscitation. Ea_dyn_ was related with the %Osc and EER [estimates: −0.101 (−0.137 to −0.064) and −9.494 (−11.964 to −7.024); *p* < 0.001, respectively]. So, the higher the Ea_dyn,_ the better the cardiovascular efficiency (lower %Osc and EER). Sepsis resulted in a reduced %Osc and EER, reflecting a better cardiovascular efficiency that was tracked by Ea_dyn_. Ea_dyn_ could be a potential index of cardiovascular efficiency during septic shock.

## Introduction

Variations in left ventricular (LV), stroke volume (SV), and arterial pulse pressure (PP) are closely related to changes in intrathoracic pressure during respiration (Pinsky, 1997). The magnitude of these changes has been used to define preload-dependency (Pinsky, 2012). Since the change in arterial PP for a change in SV has the dimension of elastance, the ratio between the PP variation (PPV) and SV variation (SVV) has been described as dynamic arterial elastance (Ea_dyn_). This parameter has been proposed as a functional measure of the arterial load (Monge Garcia et al., 2014). It arises from the simultaneous assessment of PP and SV changes during a respiratory cycle and is related to the cardiac modulation of volume as a function of pressure (Monge Garcia et al., 2014). Although Ea_dyn_ was initially defined as a variable describing central arterial tone, Ea_dyn_ is affected not only by arterial factors but also by the heart rate (HR) and the pattern of blood flow during experimental changes in arterial load conditions (Monge Garcia et al., 2017). Moreover, we have recently demonstrated a significant relationship between Ea_dyn_, ventriculo-arterial coupling, and LV mechanical efficiency (Monge Garcia et al., 2020). Accordingly, Ea_dyn_ could be considered as a composite index reflecting the interaction between both arterial and cardiac factors and potentially providing valuable information regarding cardiovascular performance and ventriculo-arterial coupling.

Although the primary function of the cardiovascular system is to deliver hydraulic energy (expressed in terms of blood flow and arterial pressure) to sustain normal physiological functions, there is an optimal combination of cardiac function and arterial system status that provides the maximum efficiency in transferring this mechanical energy from the LV to the arterial tree (Guarracino et al., 2013). Prior studies have demonstrated that this ideal combination occurs when the ventricle and the arterial tree are optimally coupled (Sunagawa et al., 1983; Asanoi et al., 1989).

Septic shock has a profound impact on macrohemodynamics and also the microcirculation (De Backer et al., 2002; Guarracino et al., 2014). Macrocirculatory derangements in septic shock have been characterized by a combination of a variable degree of cardiac dysfunction and a profound vasoplegia (Vieillard-Baron and Cecconi, 2014). These alterations are usually the expression of a ventricular-arterial uncoupling associated with a sepsis-induced cardiovascular inefficiency (Guarracino et al., 2014, 2019).

Since Ea_dyn_ has been shown to be affected by both arterial and cardiac factors, we hypothesized that sepsis-induced hemodynamic cardiovascular dysfunction could affect Ea_dyn_. We, therefore, aimed to determine the effects of endotoxic septic shock on Ea_dyn_, and the impact of hemodynamic resuscitation.

## Materials and Methods

Eighteen New-Zealand rabbits (weight 2.5 ± 0.1 kg), supplied by the Reproduction Laboratory of the University of Cadiz, were maintained at a controlled temperature (23°C) in individual cages on a 12 h light/dark and fed with a standard rabbit chow diet and water up to the time of experimental procedures. Animals were allowed to acclimatize to the laboratory for 1 week before the beginning of the experiments. All methods and protocols were approved (project 06-04-15-230) by the Ethical Committee for Animal Experimentation of the School of Medicine of the University of Cadiz (license 07–9604) and the Dirección General de la Producción Agrícola y Ganadera of the Junta de Andalucía. Animal care and use procedures conformed to national and European Union regulations and guidelines (Spanish Royal Decree 53/2013 and EU Directive 2010/63/EU). The ARRIVE guidelines were used for the elaboration of this manuscript (Group, 2010).

### Anesthesia and Instrumentation

Animals were premedicated with an intramuscular dose of xylazine (10 mg⋅kg^–1^) and ketamine (40 mg⋅kg^–1^). Then they were tracheotomized and their lungs mechanically ventilated in volume-controlled mode (Servo 900c, Siemens-Elema, Solna, Sweden), with 8 ml⋅kg**^–^**^1^ of tidal volume, positive end-expiratory pressure of 0 cmH_2_O, an inspiration to expiration ratio of 1:2, inspired oxygen fraction of 0.6, and a respiratory rate of 35–40 breaths⋅min**^–^**^1^ adjusted to maintain an end-tidal CO_2_ between 35 and 45 mmHg. The right internal jugular vein was catheterized for continuous sedation with ketamine (10–40 mg⋅kg^–1^⋅h^–1^) and midazolam (1–3 mg⋅kg^–1^⋅h^–1^). The muscular blockade was maintained with a rocuronium bromide infusion (0.6–1.2 mg⋅kg^–1^⋅h^–1^) (Quesenberry and Carpenter, 2012). Adequacy of anesthesia before neuromuscular blockade and through the experiment was evaluated by the absence of any significant blood pressure and/or heart rate change in response to an external noxious stimulus. A Ringers lactate solution (6 ml⋅kg^–1^⋅h^–1^) was administered as a maintenance fluid therapy. Animal temperature was continuously monitored by a rectal probe and maintained between 38 and 40°; using a heating pad. A 22G sterile polyethylene catheter was introduced into the right femoral artery and connected to a pressure transducer (TruWave, Edwards Lifesciences LLC, Irvine, CA, United States) zeroed against atmospheric pressure. Optimal damping of the arterial waveform was checked by a square wave test. The left femoral vein was used to administer vasoactive drugs and fluid bolus.

### Hemodynamic Monitoring

A pediatric esophageal Doppler probe (KDP72; CardioQ Combi, Deltex Medical, Chichester, United Kingdom) was introduced into the esophagus until obtaining the best outline and maximal peak velocity of aortic blood waveform. Cardiac output (CO) was calculated using the minute distance of aortic blood flow, which represents the distance traveled by a column of blood in 1 min and is calculated by the Doppler system as the product of HR and the velocity-time integral of the aortic flow waveform. The peak velocity and maximum acceleration of blood flow were used as indexes of the left ventricular systolic function (Sabbah et al., 1986; Wallmeyer et al., 1988). The arterial pressure signal was transferred from the multi-parametric monitor (S/5, Datex-Ohmeda, Helsinki, Finland) to the Doppler system and automatically synchronized with the aortic blood flow waveform for pressure-flow analysis.

### Evaluation of Arterial Load

A 3-element Windkessel model was used for characterizing the arterial system (Westerhof et al., 2009), consisting of systemic vascular resistance [SVR = mean arterial pressure (MAP)/CO]; arterial compliance (C_art_ = stroke volume / arterial pulse pressure) (Remington et al., 1948) and characteristic impedance (Zc). Zc represents the arterial input impedance in the absence of arterial wave reflections (Nichols and O’Rourke, 2005). Assuming that arterial reflections are negligible during early systole (Milnor, 1989; Nichols and O’Rourke, 2005), Zc was estimated as the slope of the early ejection pressure-flow relationship, using the ratio between the maximum of the first derivate of pressure and flow (Li, 1986; Lucas et al., 1988).

The effective arterial elastance (Ea) was used as a lumped parameter of arterial load accounting for both mean and pulsatile components (Kelly et al., 1992a):

$$E\,a=R_{T}/\left[t_{s}+\tau \times \left(1-{\text{e}}^{-t\,d}\,/\,\tau \right)\right]$$

Where R_T_ is the total mean vascular resistance (SVR + Zc), t_s_ and t_d_ are systolic and diastolic periods, respectively, and τ the diastolic time constant (τ = SVR × C_art_) (Kelly et al., 1992a).

### PPV, SVV, and Dynamic Arterial Elastance

**FIGURE 1:**
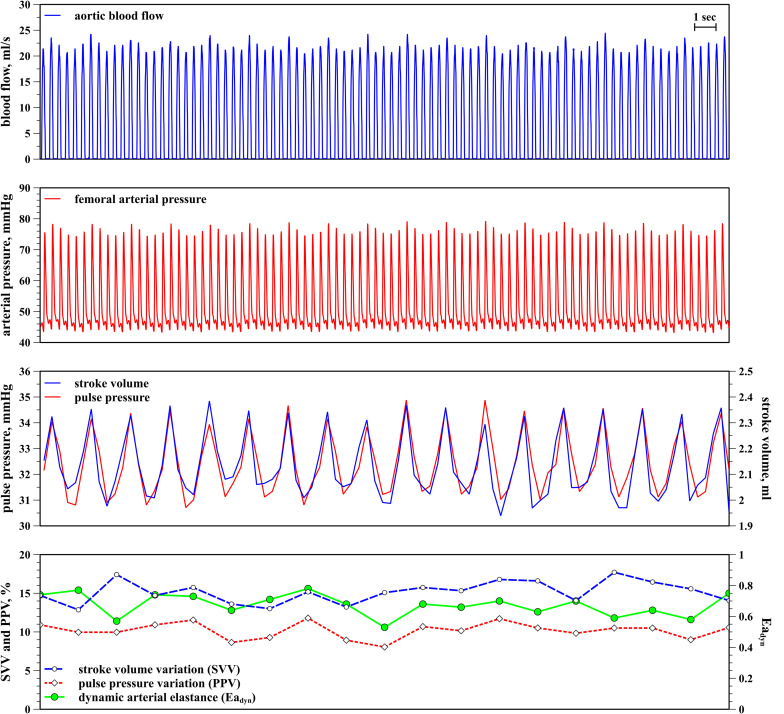
Calculation of pulse pressure variation (PPV), stroke volume variation (SVV), and dynamic arterial elastance (Ea_dyn_). From top to bottom: aortic blood flow (blue); femoral arterial pressure (red); beat-to-beat stroke volume and arterial pulse pressure (pink and dark blue); stroke volume variation (SVV, dotted red line), pulse pressure variation (PPV, blue dashed line) and dynamic arterial elastance (Ea_dyn_, solid green line).

PPV was calculated as the percentage changes in arterial pulse pressure during a ventilatory cycle as [(PPmax – PPmin)/(PPmax + PPmin)/2] × 100, where PPmax and PPmin represent the maximal and minimal arterial pulse pressure, respectively. The calculation of SVV was performed similarly. SVmax and SVmin were calculated by integrating the systolic component of aortic blood flow waveform, whereas PPV was derived from the femoral arterial pressure waveform. PPV and SVV values were simultaneously analyzed and averaged during 1 min using a custom Excel macro (Microsoft Corporation, Redmond, WA, United States) (Figure 1). Ea_dyn_ was calculated as beat-to-beat PPV/SVV averaged during 1 min (Monge Garcia et al., 2011).

### Left Ventricular Energetics

**FIGURE 2:**
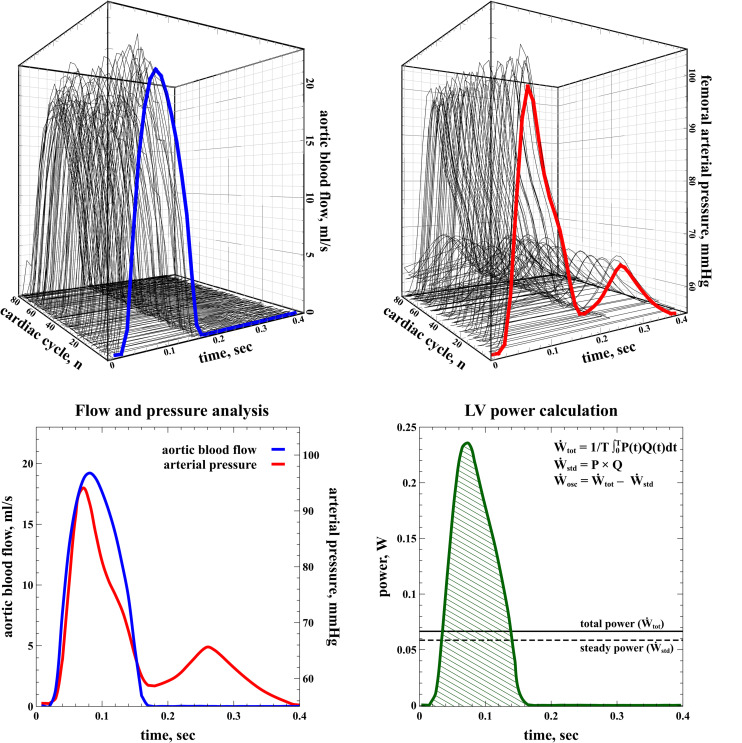
Calculation of left ventricular energetics. Aortic blood flow (top left, blue line) and arterial pressure waveforms (top right, red line) were ensembled-averaged (bottom, left) for analysis. The total LV power (Ẇ_to__t_) transferred to the systemic circulation was calculated as the time-averaged integral of the instantaneous product of pressure and flow during the whole cardiac period (bottom right, green shaded area). The difference between total power (Ẇ_to__t_) and steady power (Ẇ_std_, solid line) represents the extra expenditure of energy imposed by the pulsatile nature of the heart, i.e., the oscillatory power (Ẇ_osc_, dashed line).

Left ventricular energetics were analyzed from the instantaneous pressure and flow recordings. Aortic blood flow and femoral arterial pressure waveforms were recorded simultaneously during at least 20 s at 180 Hz and ensemble-averaged, foot-to-foot aligned using the maximum of the second derivative (Vardoulis et al., 2013), and interpolated to the duration of the cardiac cycle to provide a representative waveform for analysis. An illustrative example of the signal processing is shown in Figure 2. The contribution of kinetic energy was considered negligible and not included in the analysis (Milnor, 1989).

The left ventricular (LV) total power (Ẇ_to__t_) transferred to the systemic circulation was calculated as the time-averaged integral of the instantaneous product of blood pressure (P) by flow (Q) during the whole cardiac period (T):

$${\dot{\text{W}}}_{t\,o\,t}=\frac{1}{T}\,\int_{0}^{T}P\,\left(t\right)\,Q\,\left(t\right)\,dt$$

The product of mean pressure by mean flow, or steady power (Ẇ_std_), corresponds to the energy maintaining forward blood flow and represents the fraction of Ẇ_to__t_ useful for organ perfusion (O’Rourke, 1967; Milnor, 1989; Cholley and Le Gall, 2016).

$${\dot{\text{W}}}_{s\,t\,d}=\bar{P}\times \bar{Q}$$

The oscillatory power (Ẇ_osc_) refers to the energy lost in pulsatile phenomena due to cardiac contractions:

$${\dot{\text{W}}}_{o\,s\,c}={\dot{\text{W}}}_{t\,o\,t}-{\dot{\text{W}}}_{s\,t\,d}$$

### Cardiovascular Efficiency

The oscillatory power fraction (%Osc) represents the portion of Ẇ_to__t_ wasted in oscillatory power and quantifies the efficiency with which the external mechanical power was transferred into useful work from the LV to the arterial system (O’Rourke, 1967; Berger et al., 1995; Cholley and Le Gall, 2016). Therefore, the lower the %Osc, the more efficiently LV external work is delivered to the arterial system and converted into useful work for creating blood flow. %Osc has been used as a measure of the optimization of ventriculo-arterial coupling (O’Rourke, 1967; Cholley and Le Gall, 2016).

$$\%Osc=\frac{{\dot{\text{W}}}_{o\,s\,c}}{{\dot{\text{W}}}_{t\,o\,t}}\times 100$$

We also calculated the LV power necessary for generating one unit cardiac output for a given arterial load, as the energy efficiency ratio (EER) (Berger et al., 1995; Grignola et al., 2007):

$$E\,E\,R={\dot{\text{W}}}_{t\,o\,t\,a\,l}/C\,O$$

Therefore, the lower the EER, the lower the energy required for generating blood flow for a given LV afterload.

### Experimental Protocol

**FIGURE 3:**
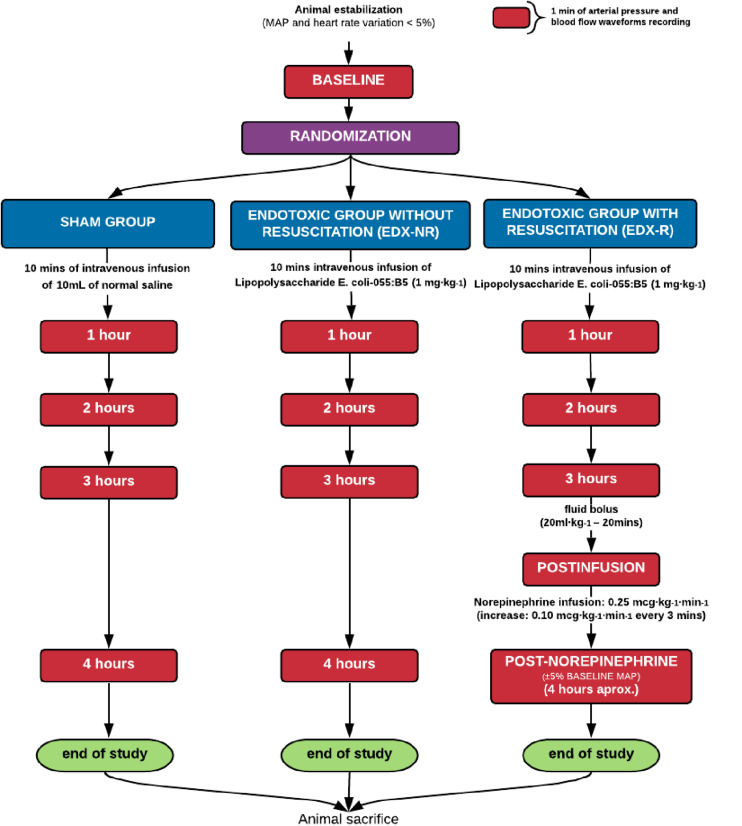
Schematic representation of the study protocol.

After completion of the surgical procedures, animals were allowed to stabilize MAP and HR (variation < 5%) at least for 10 minutes. After that, they were assigned using a computer-generated random sequence to three groups (6 animals each): a sham-operated group (SHAM), a septic group (EDX), and a septic group with hemodynamic resuscitation (EDX-R). In septic animals, a purified lipopolysaccharide (LPS) prepared from Escherichia coli serotype 055:B5 (Sigma Chemical, St. Louis, MO) was intravenously infused over 10 min through the femoral vein (1 mg⋅kg**^–^**^1^ diluted in normal saline for a total volume of 8 ml, and flushed by 2 ml of normal saline to ensure a complete delivery). The dose and rate of LPS administration were previously established on a pilot experiment with 8 animals, in which the dose was varied from 1 to 2 mg⋅kg**^–^**^1^. SHAM animals received an equivalent amount of normal saline. Three hours after LPS infusion, animals in the EDX-R group received a fluid bolus of 20 ml⋅kg**^–^**^1^ for 10 min. A norepinephrine infusion started at 0.25 mcg⋅kg**^–^**^1^⋅min**^–^**^1^ was started after fluid administration if MAP was below the baseline level. Norepinephrine was increased by 0.10 mcg⋅kg**^–^**^1^⋅min**^–^**^1^ every 3 min until reaching a MAP value similar to the baseline level (±5% deviation). Hemodynamic measurements, aortic blood flow, and arterial pressure waveforms were recorded a least during 1 min at baseline and every hour up to 4 h after LPS or placebo administration. In EDX-R animals, measurements after fluid bolus (post-infusion) and norepinephrine infusion (which corresponds to 4 h after LPS infusion) were also obtained. After completion of the study protocol, animals were euthanized using a lethal dose of intravenous potassium chloride under deep anesthesia. Animal death was confirmed by verification of the absence of blood flow and arterial pressure tracings. A schematic description of the study protocol is shown in Figure 3.

### Statistical Analysis

Data are expressed as the mean ± SD or median (25th to 75th interquartile). The normality of data was checked by the Shapiro Wilk test. Differences between groups at baseline were performed using a one-way analysis of variance (ANOVA), and differences over time were assessed by two-way mixed ANOVA for repeated measurements. The Greenhouse–Geisser correction was used when the Mauchly test detected violation of sphericity. Whenever a significant interaction was found, pairwise comparison between groups was performed using a one-way ANOVA with the Tukey-Kramer test. Mixed-effect regression analyses were used to evaluate the relationship between Ea_dyn_ (the dependent variable) and arterial load, cardiac function variables, cardiac energetics, and indexes of cardiovascular efficiency (%Osc and EER) in septic shock animals (EDX and EDX-R groups). Models were constructed using individual animals and experimental groups as subjects for random factors, and experimental stages as repeated measurements with a heterogeneous first-order autoregressive covariance structure. This allowed us to consider the correlation between subjects and non-constant variability over time, which are not considered by the standard linear regression analysis. Model selection was based on the corrected Akaike’s Information Criteria, in which lower scores indicate superior fit (Fitzmaurice et al., 2011). Model parameters were estimated via the restricted maximum likelihood method, and the estimated fixed effect of each parameter quantified by using estimated value and standard error (SE). A *P* < 0.05 was considered statistically significant. All statistical analyses were two-tailed and performed using MedCalc Statistical Software version 19.1 (MedCalc Software bvba, Ostend, Belgium^1^; 2019) and SPPS (SPSS 21, SPPS Inc., Chicago, IL).

## Results

### Hemodynamic Profile of Experimental Endotoxic Shock and Effects of Hemodynamic Resuscitation

**FIGURE 4:**
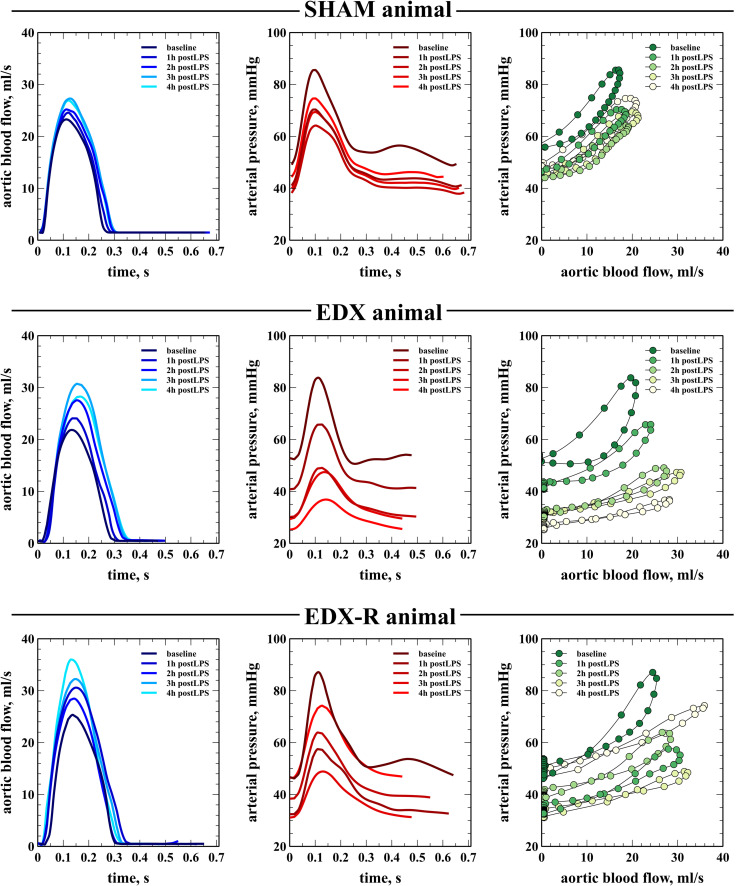
Evolution of arterial pressure-flow relationship. Example of the evolution of blood flow (left column) and the arterial pressure waveform (middle column) and the pressure-flow loops (right column) in three animals from sham-operated group (SHAM), endotoxic group (EDX), and hemodynamic-resuscitated endotoxic group (EDX-R) during the study. In this latter group, 4h stage refers to after both fluid bolus and norepinephrine infusion. Pressure-flow loops were constructed using data from the shown aortic blood flow and arterial pressure waveforms on each experimental stage.

**FIGURE 5:**
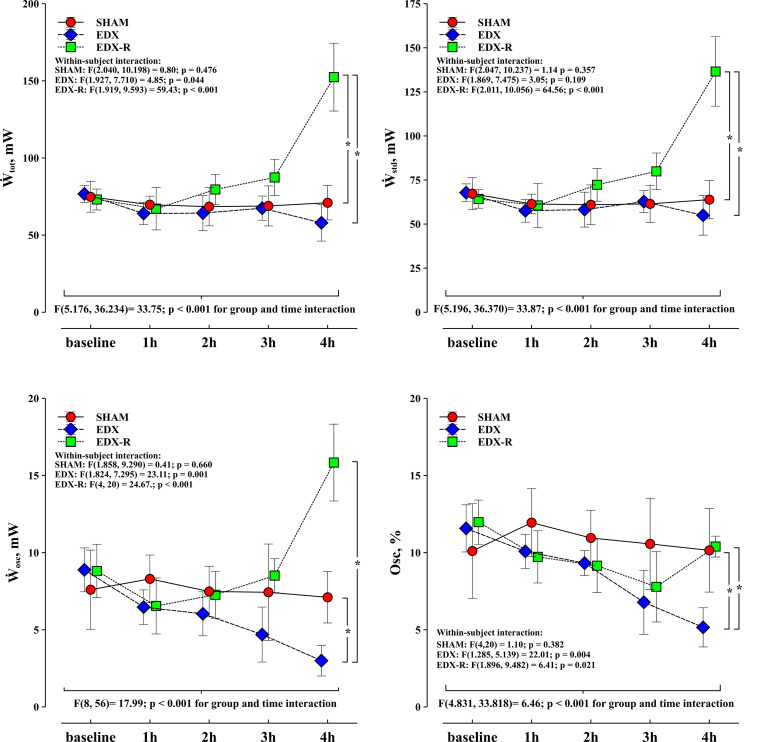
Evolution of left ventricular energetics. Changes in left ventricular total work (Ẇ_to__t_), steady power (Ẇ_std_), oscillatory power (Ẇ_osc_) and oscillatory power fraction (Osc) in the three experimental groups: Experimental arms: sham-operated group (SHAM), resuscitated endotoxic group (EDX), and hemodynamic-resuscitated group (EDX-R). Values are presented as mean ± SD. Differences in time course between groups: *p < 0.05.

**FIGURE 6:**
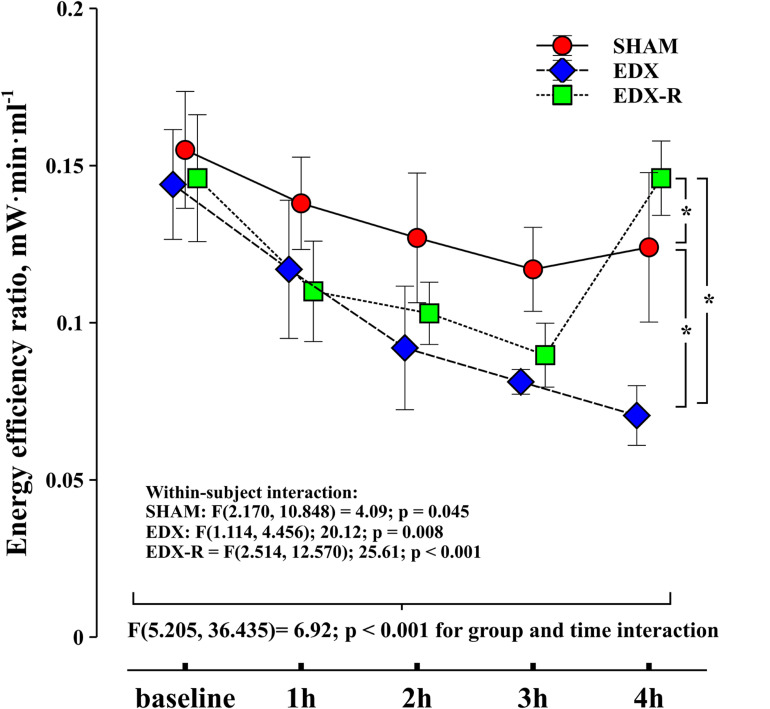
Evolution of energy efficiency ratio (EER). Experimental arms: sham-operated group (SHAM), resuscitated endotoxic group (EDX), and hemodynamic-resuscitated group (EDX-R). Values are presented as mean ± SD. Differences in time course between groups: *p < 0.05.

There were no significant differences between groups in all studied variables at baseline. The infusion of LPS resulted in a hyperdynamic hemodynamic profile with a progressive increase in CO secondary to a positive chronotropic response, a significant reduction in mean arterial pressure (35%), and a decreased arterial load (Table 1). This phenomenon was characterized by a progressive flattening of the pressure-flow relationship (Figure 4). Systolic function, as assessed by peak velocity of aortic blood flow, significantly increased in endotoxic shock animals. Although all experimental groups showed a significant decrease in the evolution of arterial load, the group and time interaction analysis showed that the changes in C and Zc were more pronounced in the EDX group when compared with the SHAM animals (Table 2). LPS reduced Ẇ_osc_ and slightly Ẇ_to__t_, but did not affect Ẇ_std_, which resulted in a better cardiovascular efficiency according to a lower Ẇ_osc/_Ẇ_to__t_ ratio (%Osc) and EER (Figures 5, 6).

**TABLE 1:** Global hemodynamic changes during the experiment.

Variables	Baseline*	1 h	2 h	3 h	4 h^*a*^	Time interaction	Group interaction	Group and time interaction
**CO, l/min**								
SHAM	0.49 ± 0.06	0.51 ± 0.03	0.54 ± 0.03	0.58 ± 0.05	0.59 ± 0.10	0.057	vs. EDX^†^	<0.001
EDX	0.54 ± 0.06	0.56 ± 0.07	0.70 ± 0.07	0.83 ± 0.07	0.81 ± 0.09	<0.001	vsEDX-R^†^	
EDX-R	0.51 ± 0.08	0.61 ± 0.10	0.77 ± 0.07	0.99 ± 0.14	1.04 ± 0.12	< 0.001	vsSHAM^†^	
**SV, ml**								
SHAM	2.7 ± 0.4	2.7 ± 0.3	2.9 ± 0.4	3.1 ± 0.4	3.0 ± 0.6	0.176		0.147
EDX	3.0 ± 0.5	2.8 ± 0.5	3.2 ± 0.7	3.5 ± 0.8	3.5 ± 0.7	0.060		
EDX-R	3.0 ± 0.4	3.4 ± 0.5	3.6 ± 0.5	4.3 ± 0.6	4.1 ± 0.6	0.005		
**SAP, mmHg**								
SHAM	87 ± 13	76 ± 8	69 ± 10	65 ± 7	61 ± 6	0.010	vs. EDX^†^	<0.001
EDX	82 ± 11	62 ± 11	50 ± 11	42 ± 4	42 ± 5	0.002	vs. EDX-R^†^	
EDX-R	85 ± 13	61 ± 11	57 ± 7	50 ± 8	77 ± 10	< 0.001	vsSHAM	
**DAP, mmHg**								
SHAM	53 ± 6	46 ± 6	43 ± 7	40 ± 4	38 ± 3	0.006	vs. EDX^†^	<0.001
EDX	48 ± 6	41 ± 7	32 ± 6	27 ± 3	26 ± 3	0.007	vs. EDX-R	
EDX-R	48 ± 7	37 ± 5	35 ± 3	29 ± 3	47 ± 6	<0.001	vsSHAM^†^	
**MAP, mmHg**								
SHAM	63 ± 7	55 ± 6	51 ± 8	47 ± 5	46 ± 3	0.006	vs. EDX^†^	<0.001
EDX	57 ± 7	47 ± 8	38 ± 8	34 ± 1	30 ± 3	0.007	vs. EDX-R^†^	
EDX-R	58 ± 8	45 ± 7	42 ± 4	38 ± 3	59 ± 5	<0.001	vsSHAM^†^	

*Data are presented as mean ± standard deviation. CO, cardiac output; DAP, diastolic arterial pressure; HR, heart rate; MAP, mean arterial pressure; SAP, systolic arterial pressure; SV, stroke volume; SHAM, sham-operated group; EDX, non-resuscitated septic rabbits; EDX-R, resuscitated septic rabbits. ^∗^Baseline values were similar between different experimental groups. ^*a*^In the EDX-R group, 4h refers after both fluid bolus and norepinephrine infusion. ^†^p < 0.05 for between-group effects (Tukey-Kramer).*

**TABLE 2:** Arterial and cardiac variables during different stages of the experiment.

Variables	Baseline*	1h	2h	3h	4h^*a*^	Time interaction	Group interaction	Group and time interaction
**Arterial load variables**								
**Ea, mmHg/ml⋅10^–1^**								
SHAM	0.83 ± 0.18	0.52 ± 0.09	0.43 ± 0.09	0.34 ± 0.04	0.36 ± 0.11	0.001		0.769
EDX	0.67 ± 0.29	0.39 ± 0.16	0.22 ± 0.08	0.16 ± 0.02	0.14 ± 0.01	0.021		
EDX-R	0.68 ± 0.23	0.31 ± 0.09	0.23 ± 0.05	0.16 ± 0.04	0.24 ± 0.05	0.001		
**C, ml/mmHg**								
SHAM	0.08 ± 0.02	0.09 ± 0.01	0.11 ± 0.03	0.13 ± 0.02	0.13 ± 0.02	0.006	vs. EDX^†^	0.001
EDX	0.09 ± 0.02	0.13 ± 0.03	0.19 ± 0.05	0.23 ± 0.04	0.31 ± 0.04	<0.001	vsEDX-R	
EDX-R	0.08 ± 0.02	0.15 ± 0.04	0.17 ± 0.06	0.21 ± 0.06	0.14 ± 0.03	<0.001	vsSHAM^†^	
**Zc, dyn⋅s⋅cm**^–^**^5^⋅10^3^**								
SHAM	0.81 ± 0.24	0.70 ± 0.17	0.58 ± 0.17	0.49 ± 0.15	0.52 ± 0.20	0.013	vs. EDX^†^	0.020
EDX	0.84 ± 0.13	0.52 ± 0.11	0.35 ± 0.06	0.23 ± 0.03	0.18 ± 0.04	0.001	vs. EDX-R	
EDX-R	0.85 ± 0.22	0.47 ± 0.10	0.39 ± 0.08	0.31 ± 0.12	0.45 ± 0.07	0.001	vs. SHAM	
**SVR, dyn⋅s⋅cm**^–^**^5^⋅10^3^**								
SHAM	10.55 ± 1.98	6.52 ± 1.01	5.72 ± 0.93	4.85 ± 0.33	4.87 ± 1.01	<0.001		0.535
EDX	8.66 ± 2.18	5.25 ± 1.68	3.25 ± 0.97	2.48 ± 0.19	2.25 ± 0.12	0.004		
EDX-R	9.37 ± 2.52	4.52 ± 1.04	3.31 ± 0.45	2.32 ± 0.35	3.42 ± 0.50	<0.001		
**Cardiac variables**								
**HR, beats/min**								
SHAM	180 ± 28	187 ± 26	191 ± 24	188 ± 21	197 ± 24	0.039	vs. EDX^†^	<0.001
EDX	182 ± 20	202 ± 22	222 ± 29	243 ± 39	237 ± 39	0.014	vs. EDX-R	
EDX-R	169 ± 17	178 ± 15	217 ± 40	234 ± 41	256 ± 18	0.004	vs. SHAM^†^	
**Accel, m/s^2^**								
SHAM	6.47 ± 0.87	6.55 ± 0.47	6.58 ± 0.54	7.09 ± 0.58	7.02 ± 1.07	0.303		0.057
EDX	6.41 ± 0.69	6.19 ± 0.84	6.81 ± 0.39	7.29 ± 1.00	6.70 ± 0.77	0.107		
EDX-R	6.95 ± 0.81	6.87 ± 0.92	7.51 ± 0.59	8.93±.03	8.96 ± 1.42	0.018		
**PV, ms/s**								
SHAM	92.2 ± 15.6	94.1 ± 10.4	97.0 ± 11.9	103.8 ± 14.3	101.6 ± 17.5	0.154	vs. EDX^†^	<0.001
EDX	98.3 ± 8.9	101.1 ± 17.9	115.8 ± 22.3	128.5 ± 24.3	123.8 ± 26.0	0.007	vs. EDX-R^†^	
EDX-R	97.8 ± 13.4	108.1 ± 17.4	123.3 ± 6.9	150.5 ± 13.6	168 ± 16.5	<0.001	vsSHAM^†^	

*Data are presented as mean ± standard deviation. Accel, mean acceleration of aortic blood flow; C, net arterial compliance; Ea, effective arterial elastance; HR, heart rate; PV = peak velocity of aortic blood flow; SVR, systemic vascular resistance; Zc, characteristic impedance; SHAM, sham-operated group; EDX, septic group without hemodynamic resuscitation; EDX-R, hemodynamic resuscitated septic group. ^∗^Baseline values were similar between different experimental groups. ^*a*^In the EDX-R group, 4h refers after hemodynamic resuscitation (both fluid bolus and norepinephrine infusion). ^†^p < 0.05 for between-group effects (Tukey-Kramer).*

The effects of hemodynamic resuscitation in the EDX-R group are summarized in Table 3. Fluid bolus increased CO and MAP by 19 and 18%, respectively, although MAP remained significantly lower than the baseline value (58 ± 8 vs. 45 ± 4 mmHg; *p* = 0.011). Norepinephrine was therefore required in all EDX-R animals to restore MAP to a similar value that baseline levels (58 ± 8 vs. 59 ± 5 mmHg; *p* = 0.504). Overall, hemodynamic resuscitation resulted in a higher cardiac energetic cost and lower cardiovascular efficiency (higher EER and %Osc).

**TABLE 3:** Effects of fluid bolus and norepinephrine in hemodynamic, arterial variables and cardiac energetics in resuscitated septic animals (EDX-R group).

	3 h post-LPS	After fluid bolus (20 mL/Kg)	After norepinephrine (4 h post-LPS)
**Hemodynamic variables**			
CO, l/min	0.99 ± 0.14	1.16 ± 0.09*	1.04 ± 0.12^*†^
SV, ml	4.3 ± 0.6	4.7 ± 0.6*	4.1 ± 0.6
SAP, mmHg	50 ± 8	61 ± 10*	77 ± 10*^†^
DAP, mmHg	29 ± 3	35 ± 2*	47 ± 6*^†^
MAP, mmHg	38 ± 3	45 ± 4*	59 ± 5*^†^
SVV, %	22 ± 4	20 ± 3	24 ± 3^†^
PPV, %	30 ± 7	19 ± 7*	21 ± 6*
Ea_*dyn*_	1.38 ± 0.32	0.93 ± 0.24*	0.87 ± 0.15*
**Arterial variables**			
Ea, mmHg/ml⋅10^–1^	0.16 ± 0.04	0.15 ± 0.03	0.24 ± 0.05
C, ml/mmHg	0.21 ± 0.06	0.19 ± 0.07	0.14 ± 0.03
Zc, dyn⋅s⋅cm^–5^⋅10^3^	0.31 ± 0.12	0.35 ± 0.13	0.45 ± 0.07
SVR, dyn⋅s⋅cm^–5^⋅10^3^	2.32 ± 0.35	2.32 ± 0.27	3.42 ± 0.50*^†^
**Cardiac variables**			
HR, beats/min	234 ± 41	251 ± 25	256 ± 18
Accel, m/s^2^	8.93 ± 1.03	9.91 ± 0.63	8.96 ± 1.42
PV, m/s	150.5 ± 13.6	171.7 ± 11.7*	168 ± 16.5^†^
**Cardiac energetics**			
Ẇ_*to*__*t*_, mW	87 ± 12	128 ± 19*	152 ± 22*^†^
Ẇ_*std*_, mW	80 ± 10	115 ± 16*	137 ± 20*^†^
Ẇ_*osc*_, mW	7 ± 2	13 ± 4	16 ± 2*
%Osc, %	9 ± 2	10 ± 2	10 ± 1*
EER, mW⋅min⋅ml^–1^	0.09 ± 0.01	0.11 ± 0.01	0.15 ± 0.01*^†^

Data are presented as mean ± standard deviation. Accel, maximal acceleration of aortic blood flow; C, net arterial compliance; CO, cardiac output; DAP, diastolic arterial pressure; Ea, effective arterial elastance; Ea_*dyn*_, dynamic arterial elastance; EER, energy efficiency ratio (Ẇ_*to*__*t*_/CO); HR, heart rate; LPS, lipopolysaccharide; MAP, mean arterial pressure; PPV, arterial pulse pressure variation; PV, peak velocity of aortic blood flow; SAP, systolic arterial pressure; SV, stroke volume; SVV, stroke volume variation; SVR, systemic vascular resistance; Ẇ_*osc*_, left ventricular oscillatory power; Ẇ_*std*_, left ventricular steady power; Ẇ_*to*__*t*_, left ventricular total power; Zc, characteristic impedance; %Osc, oscillatory power fraction (Ẇ_*osc*_/Ẇ_*to*__*t*_). *p < 0.05 vs. 3h postLPS stage, ^†^p < 0.05 vs. After fluid bolus stage (Bonferroni corrected).

### Evolution of PPV, SVV, and Ea_dyn_

**FIGURE 7:**
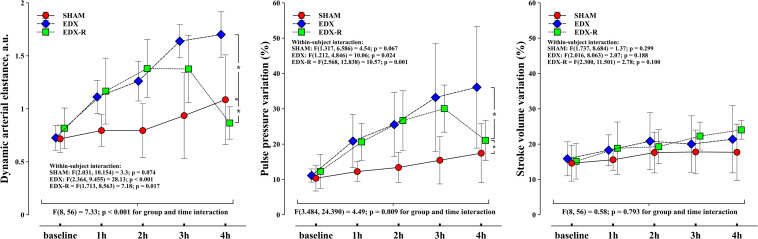
Evolution of dynamic arterial elastance, pulse pressure variation (PPV) and stroke volume variation (SVV). Experimental arms: sham-operated group (SHAM), resuscitated endotoxic group (EDX), and hemodynamic-resuscitated group (EDX-R). Values are presented as mean ± SD. Differences in time course between groups: *p < 0.05.

Figure 7 shows the development of Ea_dyn_, PPV, and SVV among different experimental groups. Ea_dyn_ increased in both EDX and EDX-R groups because of a significant increase in PPV but decreased in EDX-R animals after fluid administration (Table 3). The time course of Ea_dyn_ did not reach statistical significance in SHAM animals.

### Relationship Between Ea_dyn_, Arterial and Cardiac Factors During Septic Shock and Hemodynamic Resuscitation

When analyzing the relationship of different variables of arterial load, cardiac function, cardiac energetics, and cardiovascular efficiency indexes on Eadyn on endotoxic septic animals (EDX and EDX-R), the linear-mixed regression analysis revealed that Ea_dyn_ were related to both arterial and cardiac factors (Table 4). The overall impact of LV afterload, as assessed by Ea, was inversely associated with Ea_dyn_ (estimate: −1.082; 95% confidence interval (CI): −1.417 to −0.748; *p* < 0.001), being Zc the only component of arterial load associated to Ea_dyn_ (Table 4). On the other hand, among cardiac function variables, only heart rate was positively related to Ea_dyn_. When a linear-mixed model was used with Zc and HR as fixed effects and Ea_dyn_ as the dependent variable, their association with Ea_dyn_ remained significant, and the coefficient of determination between predicted values and Ea_dyn_ was *r*^2^ = 0.67.

**TABLE 4:** Mixed-effects regression model for the interaction between Ea_*dyn*_ and arterial load, cardiac, cardiac energetics and cardiovascular efficiency indexes in endotoxic shock animals (EDX and EDX-R groups).

Fixed effects	β estimate	95% confidence interval	*p*-value
**Arterial load variables**			
SVR, dyn⋅s⋅cm^–5^⋅10^3^	0.009	−0.056 to 0.075	0.770
C, ml/mmHg	0.509	−1.534 to 2.552	0.611
Zc, dyn⋅s⋅cm^–5^⋅10^3^	−1.053	−1.896 to −0.209	0.017
**Cardiac related variables**			
Heart rate, beats/min	0.006	0.003 to 0.008	0.002
Accel, ms/s^2^	0.047	−0.044 to 0.137	0.101
PV, m/s	−0.010	−0.050 to 0.029	0.599
**Cardiac energetics variables**			
Ẇ_*std*_, W	0.017	0.007 to 0.027	<0.001
Ẇ_*osc*_, W	−0.125	−0.174 to −0.076	<0.001
**Cardiovascular efficiency variables**			
%Osc	−0.101	−0.137 to −0.064	<0.001
EER, W⋅s⋅ml^–1^	−9.494	−11.964 to −7.024	<0.001

*Accel, maximal acceleration of aortic blood flow; C, arterial compliance; Ea_*dyn*_, dynamic arterial elastance; EER, energy efficiency ratio; PV, peak velocity of aortic blood flow; SVR, systemic vascular resistance; Ẇ_*osc*_, left ventricular oscillatory work; Ẇ_*std*_, left ventricular steady work; Zc, characteristic impedance; %Osc, oscillatory power fraction. Estimates reflect the average change in Ea_*dyn*_ per unit increase of the fixed effect. Mixed-effect regression models were constructed using arterial variables (SVR, C and Zc), cardiac variables (HR, Accel and PV), cardiac energetic variables (Ẇ_*std*_ and Ẇ_*osc*_), and cardiovascular efficiency indexes (%Osc and EER) as independent variables.*

### Relationship Between Ea_dyn_, Cardiac Energetics and Cardiovascular Efficiency During Septic Shock and Hemodynamic Resuscitation

The relationship between cardiac energetics and Ea_dyn_ in septic shock animals (EDX and EDX-R) is shown in Table 4. Although no significant association between Ea_dyn_ and Ẇ_to__t_ was found (estimate: 0.003; 95%CI: −0.003 to 0.011; *p* = 0.279), its components Ẇ_std_ and Ẇ_osc_ were independently associated: while Ẇ_std_ was positively associated with Ea_dyn_, Ẇ_osc_ did so negatively. Ea_dyn_ was also related to both estimates of cardiovascular efficiency: %Osc and EER. Accordingly, the better the efficiency (i.e., the lower EER and %Osc), the higher the Ea_dyn_.

## Discussion

In this experimental animal study, Ea_dyn_ increased during endotoxic septic shock and decreased after hemodynamic resuscitation. The evolution of Ea_dyn_ was associated with both cardiac and arterial factors and was significantly associated with cardiovascular efficiency: the higher the efficiency of the cardiovascular system, the higher the Ea_dyn_.

### Relationship Between Ea_dyn_ and Cardiac and Arterial Variables

We found that heart rate and Zc, which represent the pulsatile component of the LV hydraulic workload (Milnor, 1989), were the main variables associated to Ea_dyn_ in septic animals. Moreover, when considering only HR and Zc, 67% of the actual Ea_dyn_ values could be predicted in the septic animals. However, considering that these two variables cannot account for all the Ea_dyn_ values, we cannot exclude that other factors not contemplated in our arterial load and cardiac function assessment may have impacted the observed Ea_dyn_.

These findings corroborate previous results about the compound nature of Ea_dyn_ and the impact of both cardiac and arterial factors (Monge Garcia et al., 2020). While net changes in Ea, a net measure of LV afterload, were negatively associated with Ea_dyn_, variations in heart rate did so positively (Monge Garcia et al., 2017; de Courson et al., 2019). Our results also confirm earlier observations about the impact of endotoxic septic shock on the PPV/SVV relationship. In a model of LPS-induced pneumonia in mechanically ventilated rats, Cherpanath et al. (2014) observed that the ratio PPV/SVV was higher in LPS-treated compared with untreated rats. Septic animals also showed a significant decrease in arterial tone, as reflected by an increase in net arterial compliance (Cherpanath et al., 2014). This study, together with our previous observations about the impact of changes in arterial load (Monge Garcia et al., 2017), support the notion that Ea_dyn_ does not represent a real measure of central arterial tone nor an index of the arterial stiffness, and that the concept of Ea_dyn_ as a variable describing LV afterload should be avoided (Monge Garcia et al., 2020).

### Relationship Between LV Energetics and Ea_dyn_

The force that pumps blood from the heart to the peripheral circulation is determined by an energy gradient that comprises a kinetic, gravitational, and a potential element (Burton, 1972). During a contraction, the heart works increasing potential energy and, in a much smaller proportion, kinetic energy. This work is distributed to the systemic circulation generating the energy gradient that drives blood flow through to the systemic circulation and delivers adequate transport for oxygen and nutrients to the tissues. In turn, the hydraulic work generated by the ventricle can be divided into two components: steady and oscillatory work. While Ẇ_std_ represents the work per unit time that effectively contributes to blood flow and peripheral perfusion, Ẇ_osc_ represents the unavoidable consequence of the pulsatile nature of the heart’s activity and is considered “wasted” energy (Milnor, 1989). An efficient cardiovascular system would then minimize the oscillatory component of LV mechanical work. The ratio Ẇ_osc_/Ẇ_to__t_ (i.e., %Osc) becomes then a sort of index of the efficiency of the LV mechanical power dissipation in the arterial system (O’Rourke, 1967). Moreover, as the ability of the heart as a pump depends not only on the myocardial performance but also on the physical properties of the arterial system, %Osc represents also a measure of the efficiency of the matching between the heart and the arterial system (O’Rourke, 1967; Milnor, 1989).

While in normal conditions Ẇ_osc_ accounts only about 10% of the total LV mechanical work (O’Rourke, 1967; Nichols et al., 1986), which manifests the optimal efficiency of the arterial system for buffering cardiac pulsations, factors altering arterial load or heart rate are known to affect this balance (O’Rourke, 1967; Cox, 1974). In our study, the overall %Osc at baseline was 11 ± 2%, similar to that reported in previous animal studies (O’Rourke, 1967; Cholley et al., 1995). While in SHAM animals %Osc remained unchanged, it significantly decreased during LPS infusion and increased after hemodynamic resuscitation. These modifications agree with the known relationship between Zc and HR and %Osc. Previous studies have shown that, when Zc decreases or heart rate increases, %Osc decreases (O’Rourke, 1967; Cox, 1974; Nichols et al., 1977; Laskey et al., 1985). In our study, septic animals showed a progressive and significant decrease in Zc and increased heart rate. Not surprisingly, these same factors were the main determinants of the Ea_dyn_ changes in septic animals. As Ea_dyn_ was directly related to Ẇ_std_ but inversely to Ẇ_osc_, Ea_dyn_ reflects then not only the net LV mechanical work but how this work is generated. Therefore, changes in Osc% and EER were inversely associated with Ea_dyn_ variations. Accordingly, when cardiovascular efficiency increases, so does the Ea_dyn_ (Monge Garcia et al., 2020).

Our results differ from those reported by Cholley et al. (1995) in a similar study. These authors found that a lower dose of LPS (600 μg⋅kg**^–^**^1^) resulted in a hypodynamic hemodynamic profile characterized by low cardiac output, arterial hypotension, and increased %Osc. Septic shock was also associated with a lower heart rate and higher Zc resulting from the aortic smooth muscle contraction and aortic wall edema. These discrepancies may likely reflect differences in the experimental setup, the dose of LPS, and the anesthetic regimen used (pentobarbital vs. ketamine and midazolam). However, even if our results differ in how LPS affects hemodynamics, both are coheren t on the physiological mechanisms and how these factors are known to affect %Osc (O’Rourke, 1967; Cox, 1974; Nichols et al., 1977; Kelly et al., 1992b).

### Clinical Implications

Ventriculo-arterial uncoupling and impaired LV efficiency is a frequent phenomenon in septic shock (Yan et al., 2017; Guarracino et al., 2019; Pinsky and Guarracino, 2020). The assessment of ventriculo-arterial coupling in these patients may help to determine the underlying mechanism of cardiovascular failure and to predict the effectiveness of the hemodynamic therapies (Guarracino et al., 2019). Therefore, considering the crucial role of ventriculo-arterial coupling and LV efficiency in septic shock, the continuous assessment of Ea_dyn_ as an index for monitoring cardiovascular mechanical efficiency becomes therefore evident. However, the clinical usefulness of Ea_dyn_ as an easily measured bedside tool for monitoring VAC and LV efficiency requires further validation.

### Limitations

Our evaluation of the cardiovascular efficiency was based on the analysis of the instantaneous arterial pressure-aortic blood flow relationship (Milnor, 1989). This analysis, however, does not represent the actual LV mechanical efficiency since it does not consider myocardial oxygen consumption (Suga et al., 1981). Moreover, because of our simplified assessment of LV performance, we cannot determine the actual impact of LPS on myocardial performance, which would have required the invasive measurements of LV volume and pressure. However, we have recently demonstrated that Ea_dyn_ is significantly related to ventriculo-arterial coupling and LV efficiency using the data obtained from the LV pressure-volume analysis by the classical conductance catheter technique (Monge Garcia et al., 2018). Our current results agree with the observations made in this study. Nevertheless, even if LPS infusion was associated with an improved transfer of the power from the LV to the arterial system, we cannot confirm that this condition was only related to a better myocardial contractility. As LPS can alter not only LV contractility but also loading conditions (Pinsky and Rico, 2000; Jianhui et al., 2010), the decreased arterial load in septic animals may have played an essential role in the improved power transfer efficiency (O’Rourke, 1967). A low %Osc indicates therefore that, for a given LV myocardial performance, the arterial load was relatively lower. Thus, even if %Osc does not directly describe the ventricular-arterial coupling variables, it represents an index of the efficiency of the cardiovascular system (Berger et al., 1995; Cholley and Le Gall, 2016).

## Conclusion

During experimental endotoxic shock, Ea_dyn_ changes were associated with arterial and cardiac factors and significantly related to cardiovascular efficiency: the higher the efficiency of the cardiovascular system on delivering the energy to the arterial system for sustaining blood flow, the higher the Ea_dyn_. Therefore, Ea_dyn_ may be a valuable index for monitoring cardiovascular mechanical efficiency.

## Data Availability Statement

The raw data supporting the conclusions of this article are available to any qualified researcher from the corresponding author upon reasonable request.

## Ethics Statement

The animal study was reviewed and approved by the Ethical Committee for Animal Experimentation of the School of Medicine of the University of Cadiz (license 07–9604) and the Dirección General de la Producción Agrícola y Ganadera of the Junta de Andalucía.

## Author Contributions

MMG conceived and designed the study, participated in the experiments, performed the statistical analysis, interpreted the data, and wrote the manuscript. PG, PS, MG, and AG and participated in the experiments, interpreted the data, and helped to draft the manuscript. AM and AR have made substantial contributions to the analysis and interpretation of data, have been involved in drafting the manuscript, and contributed to its critical review. MC contributed to the conception and design of the study, interpreted the data, wrote, and reviewed the manuscript. All authors read and approved the final version of the manuscript.

## Conflict of Interest

MMG has received Honoraria and/or Travel Expenses from Edwards Lifesciences and Deltex Medical. AG has received Honoraria and/or Travel Expenses from Edwards Lifesciences. AR has received Honoraria and is on the advisory board for LiDCO, Honoraria for Covidien, Edwards Lifesciences, and Cheetah. MC in the last 5 years received Honoraria and/or Travel Expenses from Edwards Lifesciences, LiDCO, Cheetah, Bmeye, Masimo, and Deltex. The remaining authors declare that the research was conducted in the absence of any commercial or financial relationships that could be construed as a potential conflict of interest.

## Footnotes

1 https://www.medcalc.org

## Abbreviations

Ea_dyn_: dynamic arterial elastance
PPV: pulse pressure variation
SVV: stroke volume variation
HR: heart rate
CO: cardiac output
SVR: systemic vascular resistance
MAP: mean arterial pressure
C: arterial compliance
Zc: characteristic impedance
Ea: effective arterial elastance
RT: total mean vascular resistance
τ: diastolic time constant
LV: left ventricular
Ẇtot: left total ventricular power
P: blood pressure
PV: peak velocity of aortic blood flow
Q: aortic blood flow
T: cardiac period
Ẇstd: LV steady power
Ẇosc: LV oscillatory power
%Osc: oscillatory power fraction
EER: energy efficiency ratio
SHAM: sham-operated group
EDX: endotoxic group without hemodynamic resuscitation
EDX-R: endotoxic group with hemodynamic resuscitation
LPS: lipopolysaccharide
ANOVA: analysis of variance
SE: standard error
CI: confidence interval.
